# Antimicrobial stewardship: from theory to reality in a resource-limited setting (and beyond)

**DOI:** 10.3389/frabi.2024.1492319

**Published:** 2024-10-21

**Authors:** Eric Ochoa-Hein, Arturo Galindo-Fraga

**Affiliations:** Hospital Epidemiology Department, Instituto Nacional de Ciencias Médicas y Nutrición Salvador Zubirán, Mexico City, Mexico

**Keywords:** anti-infective agents, anti-bacterial agents, antimicrobial stewardship, resource-limited settings, prevention and control

## Abstract

Correct use of antibiotics is paramount to present global health. Among other actions, antimicrobial stewardship emphasizes de-escalation and suspension whenever possible. Nonetheless, roadblocks can be encountered (e.g., lack of culture results or availability of specific antibiotic classes). Furthermore, in an ever-increasing interconnected world, global success relies on local success. In this perspective, a particular case study in a resource-limited setting is an example of the many difficulties encountered in the fight against antimicrobial resistance that could hamper global advancements.

## Introduction

Antibiotics are vital for human survival in contemporary times. A whole array of lifesaving medical and surgical procedures depends on their timely use, in either a prophylactic or a therapeutic way. However, rising antibiotic resistance on the global scale threatens to stop advances achieved in the past. For instance, without any intervention, a continued rise in antimicrobial resistance could cause a similar number of deaths as cancer is expected to do in the second half of this century (O’Neill, 2016). In order to succeed, the planet as a whole must embrace all possible efforts to tackle this menace; notably, we must rely on all sorts of actions that encompass human, animal and ecosystem health (McEwen and Collignon, 2018).

With regards to the use of antibiotics in human health, antibiotic stewardship programs (ASP) have been among the most useful interventions that have curbed further increases in antimicrobial resistance (especially in the hospital setting), and many success stories have been published at the local level (Huttner et al., 2014). However, despite growing evidence of the effectiveness of ASPs, many healthcare settings (especially in resource-limited settings) still struggle to successfully complete its implementation due to various roadblocks (Kakkar et al., 2020; Sihombing et al., 2023; Gulumbe et al., 2024).

In this perspective, the study of a particular case in a Mexican setting will help to show that many challenges, including high levels of antimicrobial resistance *per se*, are powerful obstacles to the fulfillment of ASPs.

## Case study

The Instituto Nacional de Ciencias Médicas y Nutrición is a public, third-level, academic, national, referral center located in Mexico City. It mostly serves adult people living in the greater metropolitan area (that spans across two neighboring states) in need of specialized medical and surgical interventions (excluding obstetric care); of interest, up to one half of the patients attended are immunosuppressed. Hospital care is provided in 168 individual or shared rooms. The Infectious Diseases department is a consulting service that has unrelentingly been promoting ASP actions during the last decade. The ASP in our setting relies heavily on the daily discussion of antibiotic prescriptions of all hospitalized patients between treating and consulting physicians, but also includes other actions such as formulary restriction and Pharmacy reminders. Additionally, frequent education programs are offered periodically to the hospital medical staff and written guidelines for initial empirical antibiotic prescriptions were recently added.

To further strengthen these tasks, the Hospital Epidemiology Department routinely takes note of all hospitalized patients administered any broad-spectrum antibiotic (especially, third- and fourth-generation cephalosporins, carbapenems, vancomycin and piperacillin/tazobactam) during at least 7 days, and meetings to discuss these cases individually are convened on a weekly basis. During these meetings, recommendations stemming from the appropriate use of antibiotics (such as prompt stopping orders, de-escalation, dose adjustments, assessment of drug-drug interactions and diagnostic stewardship) are made by the leaders (infectious diseases physicians of the Hospital Epidemiology Department) to treating and consulting physicians. The recorded data can be used afterwards for auditing purposes, which was retrospectively done in 2021 in an effort to assess the impact of the COVID-19 pandemic on prescription practices, as well as to analyze the bacterial isolates recovered from patients that were subjected to this particular surveillance system.

During 2021, a total of 687 hospitalized patients with prolonged use of broad-spectrum antibiotics were audited. Infections were mostly from intraabdominal (n=164), pulmonary (n=109), urinary (n=84), intravascular (n=73) and osteoarticular (n=59) sources. An isolate was recovered in 175 of the 687 cases (25.5%) and this was due to particular factors that occurred in our hospitalized patients during the COVID-19 pandemic (for instance, a great number of patients with intraabdominal infections were treated with antibiotics before being referred to us, and not all patients with pulmonary infections had positive cultures because viral infections predominated in hospitalized patients with pneumonia). The rate of recovery of organisms resistant to at least one antibiotic by infection source was: intraabdominal=33.5%, pulmonary=7.3%, urinary=41.7%, intravascular=43.8% and osteoarticular=23.7% The most frequently isolated microorganisms (only the first ones in case of repeated cultures) and their resistance patterns (as defined by Magiorakos et al., 2012) are shown in **Table 1**. The most frequently prescribed antimicrobials during the audited period (i.e., the number of prescriptions) were ertapenem (n=200), meropenem (n=132), vancomycin (n=131), piperacillin/tazobactam (n=115), imipenem (n=85) and third- or fourth-generation cephalosporins (n=51). The distribution of the antibiotics most frequently prescribed by type of bacteria (gram-positive, gram-negative or unknown) is shown in **Table 2**.

**Table 1:** Most frequently isolated microorganisms (n=175).

Bacterial isolate	ESBL	NDM	MDR/ XDR	Methicillin-resistant	Other*	Total
*Escherichia coli*	85 (83.3%)	5 (4.9%)	7 (6.9%)		5 (4.9%)	102 (58.3%)
*Pseudomonas aeruginosa*			17 (81.0%)		4 (19.0%)	21 (12.0%)
*Klebsiella pneumoniae*	12 (80.0%)	1 (6.7%)			2 (13.3%)	15 (8.6%)
*Enterococcus faecalis/faecium*			2 (20.0%)		8 (80.0%)	10 (5.7%)
*Staphylococcus aureus*				9 (100%)	0	9 (5.1%)
*Staphylococcus epidermidis*				8 (100%)	0	8 (4.6%)
*Acinetobacter baumannii*			2 (50.0%)		2 (50.0%)	4 (2.3%)
Other bacteria						6 (3.4%)

ESBL, extended-spectrum beta-lactamase; NDM, New Delhi metallo-beta-lactamase; MDR, multidrug-resistant; XDR, extensively drug-resistant.

*Other: a type of resistance that is not included in the classification of Magiorakos et al. (2012) or isolates with an undetermined resistance mechanism that are also not classified under any of the groups proposed by Magiorakos et al. (mainly, isolates with resistance to only one antibiotic).

**Table 2:** Distribution of the most frequent antibiotic prescriptions by type of bacteria.

Antibiotic	Gram-positive	Gram-negative	Unknown*	Total
Ertapenem	0	134	66	200
Meropenem	1	81	50	132
Vancomycin	58	0	73	131
Piperacillin/tazobactam	23	36	56	115
Imipenem	7	34	44	85
Cephalosporin (3rd/4th generation)	10	26	15	51
**Total**	99	311	304	714

*This category includes both negative cultures and patients that did not have cultures done.

Of the 687 audited cases, 176 (25.6%) were listed during two or more consecutive weeks (owing to prolonged lengths of stay), and were thus amenable to the follow-up of recommendations made in previous meetings. Recommendations were followed in 146 of those 176 cases (83.0%); nonetheless, in 136 of 146 cases (93.2%), a recommendation to continue broad-spectrum antimicrobial treatment was given (given the fact that opportunities to de-escalate or shorten treatment duration were lacking). The main reasons underlying the inability to stop or de-escalate antibiotics mostly included the following: a) technical issues in surgery (e.g., an inability to drain multiple small abscesses), b) administrative roadblocks in surgery (e.g., lack of surgical resources to operate in a safe and timely manner), c) technical issues in medicine (such as the inability to shorten treatment duration in severely ill immunosuppressed patients or to adjust treatment due to the presence of MDR/XDR isolates), and d) administrative roadblocks in medicine (e.g., lack of a narrow-spectrum recommended antibiotic in the national/local drug formulary).

## Discussion

In a set of highly selected hospitalized patients characterized by the use of broad-spectrum antibiotics during a consecutive week or more in a Mexican center, de-escalation or early stopping opportunities proved to be particularly complicated due to multiple factors, including the presence of highly resistant bacterial isolates.

As exemplified by the case study, there is little room for antibiotic de-escalation or stop orders given the current conditions in this referral hospital, a situation that could be present in many other healthcare settings around the world. However, opportunities for action still exist. A discussion of measures that should be helpful in resource-limited settings as ours is henceforth offered.

The economic utility of ASPs must be firmly established in low- and middle-income countries and communicated to decision makers. One could intuitively assume that ASPs are cost-saving in certain circumstances (e.g., with the avoidance of antibiotic prescriptions in patients with common colds). Furthermore, some studies (albeit not all) have conclusively concluded that ASPs are cost-effective or cost-saving (Nathwani et al., 2019; Trotter et al., 2023). Although it is tempting to extrapolate data, the truth is that there is a dearth of information for low- and middle-income countries, where situational contexts can deviate greatly from those of developed countries (Nathwani et al., 2019; Gebretekle et al., 2021; Aiesh et al., 2023).

Research in antimicrobial resistance must be supported. Data must be generated *in vivo* and microorganisms must be identified. Unfortunately, publicly funded research in resource-limited settings is scarce (Pierce et al., 2020), since health budgets are mostly devoted to medical assistance tasks. Additionally, as is the case in Mexico, many hospitals lack access to a certified microbiology laboratory capable of providing timely information on antimicrobial resistance, further restricting research opportunities. Due to competing priorities and lack of resources, many healthcare settings around the globe are unable to fulfill qualified surveillance tasks (such as prospective surveillance); as a result, a great number of outbreaks and resistant microorganisms go unnoticed.

Every healthcare institution in the world must be able to analyze its own data and create/adapt policies accordingly. What occurs in one place does not necessarily apply to others. Regional differences in resistance rates exist within and among countries (Antimicrobial Resistance Collaborators, 2022; Aronin et al., 2022). Furthermore, particular cultural and workflow issues, reflective of community values, are also present in different healthcare settings. Priorities can shift importantly from one region to another, emphasizing the need for tailored research and actions at the local level.

Even if little room for action is apparently present, every effort to put ASPs into practice must be exercised. Inaction is a powerful enemy of change and progress. Evidence has already shown that benefits can be gained when small changes are adopted, such as when duration of antimicrobial treatment is reduced (Mo et al., 2023; Vitiello et al., 2024). Therefore, it must not be assumed that an inability to avoid the use of antibiotics altogether nullifies any effort brought by small but meaningful advancements.

A multidisciplinary approach must be embraced. All kinds of medical, nurse and pharmacy practitioners with different backgrounds are allowed to prescribe antibiotics around the world. Antibiotic prescription takes place in ambulatory and hospitalized settings. Some infections can be treated by general practitioners or surgeons without the need for antibiotics (e.g., by drainage, as in skin and soft tissue abscesses). These scenarios remind us of the importance of including all relevant actors in ASPs.

Prevention policies must be the top priority in resource-limited settings. Too often, preventive actions are unforgivingly omitted. The leading causes of medical consultation in the great majority of resource-limited countries are preventable infectious diseases (especially, of respiratory and gastrointestinal origin). Evidence indicates that simple and low-cost preventive interventions, such as hand hygiene, have a big impact on the health of communities (United Nations Children’s Fund and World Health Organization, 2021). For instance, two decades algo it was demonstrated that a community intervention that focused on hand hygiene promotion in a resource-limited country halved the incidence of both respiratory and gastrointestinal diseases in children under 5 years of age (Luby et al., 2005). Of course, other actions such as vaccination (notably, against the leading viral agents that cause severe pneumonia) could avert use of antibiotics in a great number of patients.

Community engagement is vital. Hospitals are not isolated systems. One of the greatest lessons of the case study in our setting is the realization that referred patients (many of them previously treated with antibiotics) congregate in our hospital, therefore posing a risk for the continuous seeding of resistant microorganisms that could then propagate if unchecked by timely preventive measures. But the inverse also holds true: magnification of the resistance phenomenon in the hospital can overflow back to the community. It therefore follows that, in the long run, any progress in healthcare settings will be threatened if not accompanied by progress in the community. Unfortunately, in our hospital, antibiotic choices are sometimes severely limited by resistant isolates that were already acquired in the community, and are further restricted when faced with the additional types of challenges that were mentioned at the end of the case study.

Support of health systems in resource-limited settings is needed. Resistance crosses borders. Analogous to the previous discussion, microorganisms do not remain static in one region or continent. There are examples of resistant microorganisms that originated in one region and then spread to others (Dortet et al., 2014; Chakrabarti and Sood, 2021). Although it is logic and fair to hope for proactive measures in individual countries, the truth is that only international interest, cooperation and support will guarantee success for all in the future. In order for all to succeed, resource-limited settings must be particularly supported; in this regard, initiatives like the Global Antimicrobial Stewardship Accreditation Scheme (GAMSAS) will be vital for the efforts to contain antimicrobial resistance risks around the world (Gulumbe et al., 2024).

## Conclusions

There is no doubt that the conscientious use of antibiotics will literally remain a lifesaving intervention in the near future. However, this need is threatened by roadblocks shared by the majority of resource-limited countries. The case study highlights a particular situation in which the concurrence of technical and administrative challenges (including high resistance rates *per se*) limits the ability of a healthcare setting in a resource-limited country to fulfill the antimicrobial stewardship principles. In the context of a globalized world, this fact not only poses a risk at the local level, but also endangers other regions where positive results have been achieved.

## Data Availability

The raw data supporting the conclusions of this article will be made available by the authors, without undue reservation.
